# What do medical students need to know about pediatric musculoskeletal (pMSK) medicine? Defining the learning outcomes

**DOI:** 10.1186/1546-0096-10-S1-A8

**Published:** 2012-07-13

**Authors:** Sharmila Jandial, Jane Stewart, Lesley Kay, Helen E Foster

**Affiliations:** 1 Newcastle University, Newcastle Upon Tyne, Tyne and Wear, UK; 2 Newcastle upon Tyne Hospitals NHS Foundation Trust, Newcastle upon Tyne, Tyne and Wear, UK

## Purpose

Musculoskeletal problems in childhood are common, presenting to both primary care and hospital specialities. However doctors involved in the care of children report poor confidence in their pMSK clinical skills; pMSK education is infrequently included in current medical school teaching within the UK and US. The development of the pediatric Gait, Arms, Legs and Spine MSK screening examination (pGALS) aimed at medical students is an important step to improving pMSK clinical skills but requires context. Our aim was to define learning outcomes (clinical skills and knowledge) within pMSK medicine to be acquired by graduation. pMSK medicine should be taught by both pMSK specialist and non-specialist teachers; a secondary aim was to identify barriers to pMSK teaching which would inform implementation of this curriculum.

## Methods

A two-phase study was used. In Phase 1, proposals for pMSK curriculum content and barriers to pMSK teaching were generated from focus groups and interviews. Phase 2 achieved consensus on the final curriculum content using a modified Delphi process followed by group nominal technique. Participants were recruited from stakeholder groups: pediatric rheumatology and orthopedics, general and specialist pediatrics, family practice, allied health professionals and medical students. The project had full ethical approval and was funded by Arthritis Research UK.

## Results

**Figure 1:**
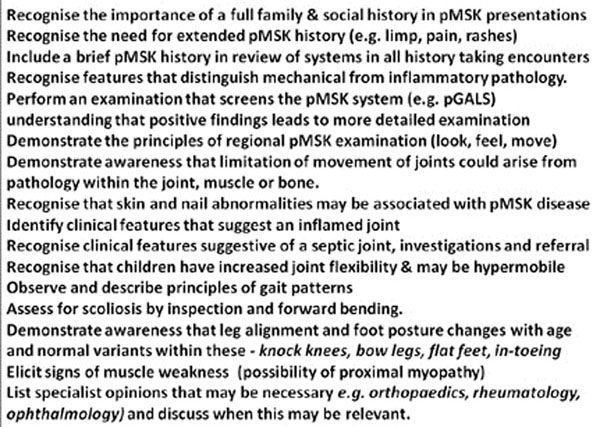
pMSK-specific learning outcomes

Phase 1 generated 60 potential learning outcomes. Consensus was achieved in Phase 2 on learning outcomes (n=47) alongside core presentations (n=8) and core conditions (n=14) to provide context. Many learning outcomes were associated with generic child health concepts (e.g. development, communication). pMSK specific outcomes (n=16) related mainly to physical examination (11/16) and could be covered by adequate teaching of pGALS. The ‘limping child’ as a core presentation covered the majority of learning outcomes within the pMSK curriculum (n=30) including “red flags” for serious illnesses and core conditions (7/14). Barriers to pMSK teaching were numerous (e.g. non-specialist teachers with low confidence and poor knowledge within pMSK medicine, teaching focussed on in-patients with under-representation of pMSK patients, time pressures on teachers and within curricula, absence of pMSK medicine within final assessments). Notably pMSK medicine was deemed to be ‘core’ for medical students by all stakeholder groups. Figure 1.

## Conclusion

This is the first consensus based content for an undergraduate pMSK curriculum involving all stakeholders within pMSK medicine. Principles specific to pMSK medicine relate to clinical skills; appropriate teaching of pGALS and the limping child is necessary. Barriers to implementation are important to address and should include improved training and support for child health teachers, access to children with pMSK problems and inclusion of valid pMSK assessments within undergraduate training.

## Disclosure

Sharmila Jandial: None; Jane Stewart: None; Lesley Kay: None; Helen E. Foster: None.

